# Impairment of HIF-1α-mediated metabolic adaption by *NRF2*-silencing in breast cancer cells

**DOI:** 10.1016/j.redox.2019.101210

**Published:** 2019-05-02

**Authors:** Sujin Lee, Steffanus Pranoto Hallis, Kyeong-Ah Jung, Dayoung Ryu, Mi-Kyoung Kwak

**Affiliations:** a Department of Pharmacy and BK21PLUS Team for Creative Leader Program for Pharmacomics-based Future Pharmacy, Graduate School of The Catholic University of Korea, 43 Jibong-ro, Bucheon, Gyeonggi-do, 14662, Republic of Korea; b Faculty of Biotechnology, Atma Jaya Catholic University of Indonesia, Jakarta, 12930, Indonesia; c Integrated Research Institute for Pharmaceutical Sciences, The Catholic University of Korea, Republic of Korea; d College of Pharmacy, The Catholic University of Korea, Republic of Korea

**Keywords:** Hypoxia, Metabolism, Autophagy, HIF-1α, NFE2L2/NRF2, Metabolome

## Abstract

Hypoxia, a common element in the tumor environment, leads to Hypoxia-Inducible Factor-1α (HIF-1α) stabilization to modulate cellular metabolism as an adaptive response. In a previous study, we showed that inhibition of the nuclear factor erythroid 2-like-2 (NFE2L2; NRF2), a master regulator of many genes coping with electrophilic and oxidative stress, elevated the level of *miR-181c* and induced mitochondrial dysfunction in colon cancer cells. In this study, we demonstrate that *NRF2*-silencing hindered HIF-1α accumulation in hypoxic breast cancer cells and subsequently suppressed hypoxia-inducible expression of glycolysis-associated glucose transporter-1, hexokinase-2, pyruvate dehydrogenase kinase-1, and lactate dehydrogenase A. HIF-1α dysregulation in *NRF2*-silenced cancer cells was associated with *miR-181c* elevation. Overexpression of *miR-181c* in breast cancer cells blocked HIF-1α accumulation and diminished hypoxia-inducible levels of glycolysis enzymes, whereas the inhibition of *miR-181c* in *NRF2*-silenced cells restored HIF-1α accumulation. In a subsequent metabolomic analysis, hypoxic incubation increased the levels of metabolites involved in glycolysis and activated the pentose phosphate pathway (PPP) in control cells. However, these elevations were less pronounced in *NRF2*-silenced cells. In particular, hypoxic incubation increased the levels of amino acids, which implies a shift to catabolic metabolism, and the increased levels were higher in control cells than in *NRF2*-silenced cells. Concurrently, hypoxia activated BCL2 interacting protein 3 (BNIP3)-mediated autophagy in the control cells and *miR-181c* was found to be involved in this autophagy activation. Taken together, these results show that hypoxia-induced metabolic changes to glycolysis, the PPP, and autophagy are inhibited by *NRF2*-silencing through *miR-181c*-mediated HIF-1α dysregulation. Therefore, targeting *NRF2*/*miR-181c* could be an effective strategy to counteract HIF-1α-orchestrated metabolic adaptation of hypoxic cancer cells.

## Graphical abstract

**Figure:**
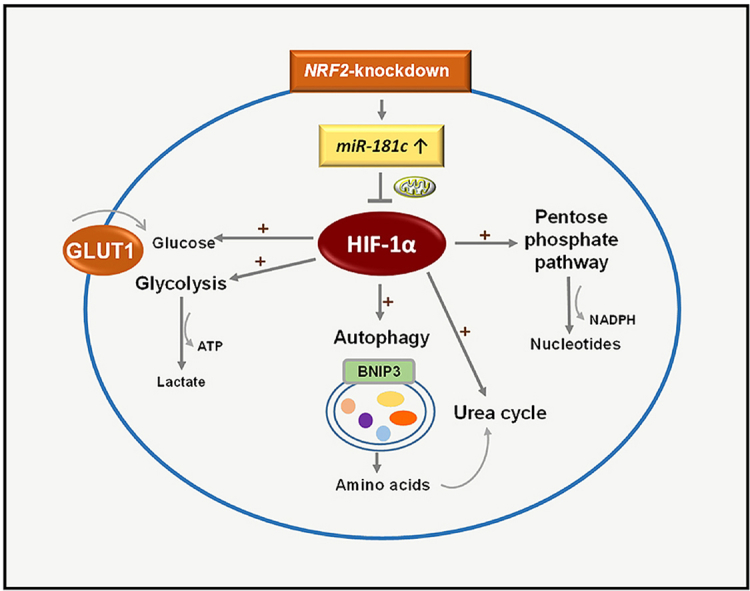

## 1 Introduction

Hypoxia-inducible factor-1 (HIF-1) senses cellular oxygen (O_2_) levels and induces an adaptive response to hypoxia by upregulating an array of genes associated with angiogenesis, cell growth, and glycolytic metabolism [1,2]. HIF-1 is composed of O_2_-regulated HIF-1α and its constitutive subunit HIF-1β [3]. In the presence of normal O_2_ levels, the stability of the HIF-1α protein is regulated by prolyl hydroxylases domain protein (PHD)-mediated hydroxylation at the Pro^402^ and Pro^564^ residues and the Von Hippel-Lindau protein (VHL)-mediated proteasomal degradation [2,4,5]. In this reaction, O_2_ is used as a substrate of PHDs and, therefore, the hypoxic condition causes inhibition of the PHD-mediated hydroxylation and VHL-mediated degradation of HIF-1α. Accumulated HIF-1α binds to hypoxia-responsive elements (HREs) on the promoter of its target genes, which are involved in aggressive behaviors of cancers, including blood vessel formation [6,7], metabolic reprogramming [8,9] and metastasis/invasion [10,11].

The modulation of metabolic pathways is critical for cancer cell survival and growth in response to environmental changes. Upon facing hypoxia, cancer cells switch their metabolism to glycolytic pathways through HIF-1α signaling [12]. First, HIF-1α accumulation directly upregulates glucose transporter-1 (GLUT1), a transporter for cellular uptake of glucose, and hexokinase-2 (HK2), an enzyme initiating glucose metabolism by phosphorylating glucose to produce glucose-6-phosphate (G6P) [13]. HIF-1α additionally inhibits mitochondrial oxidative phosphorylation and tricarboxylic cycle (TCA cycle) through the induction of pyruvate dehydrogenase kinase-1 (PDK1), an enzyme that inhibits pyruvate dehydrogenase (PDH) through phosphorylation [14]. This, in turn, represses the conversion of glycolytic pyruvate into mitochondrial acetyl-coenzyme A and thereby impairs mitochondrial oxidative phosphorylation. This leads to accumulation of pyruvate and further conversion to lactate through the enzymatic action of lactate dehydrogenase A (LDHA), which is also regulated by HIF-1α [15]. Additionally, HIF-1α elevation has been shown to benefit cancer cells by promoting anabolic metabolism, including the pentose phosphate pathway (PPP), which can provide hypoxic cancer cells with nucleotides as building blocks [16].

Autophagy is important for maintaining energy in times of nutrient deprivation [17]. The process of autophagy involves the formation of double-membrane structured autophagosomes, which fuse with lysosomes to form autolysosomes where cellular components are degraded by lysosomal enzymes [18,19]. Autophagy participates in the maintenance of basal cellular homeostasis by clearing abnormal proteins and removing injured intracellular organelles such as mitochondria [19,20]. Although autophagy is considered to be a normal cellular process maintaining cellular homeostasis, it is generally thought to act as a protective mechanism against various stress conditions such as nutrient depletion, protein aggregations, and genotoxic agents [21,22].

Nuclear factor erythroid 2-like-2 (NFE2L2; hereafter NRF2) plays a crucial role in the basal and inducible expressions of multiple cytoprotective genes in response to electrophilic and oxidative stress [23]. The cytosolic actin-binding protein Kelch-like ECH-associated protein 1 (KEAP1) primarily regulates NRF2 activity through the Culilin3-based E3 ligase-dependent degradation [24]. In the presence of reactive oxygen species (ROS) or electrophiles, NRF2-KEAP1 binding is disrupted through modification of cysteine residues of KEAP1 protein, and thus free NRF2 protein accumulates in nucleus, which leads to the transactivation of antioxidant response element (ARE)-bearing cytoprotective genes [23,24]. However, high levels of NRF2 has been beneficial to cancer cells by eliminating excess ROS, which are derived from uncontrolled energy production in cancer cells, and by facilitating tumor growth and anticancer drug metabolism [[25], [26], [27], [28]]. Moreover, NRF2 overactivation has been recognized as a factor inducing metabolic reprogramming of cancers, including the activation of the pentose phosphate pathway (PPP) and glutaminolysis [28].

We previously demonstrated that *NRF2*-silenced colon cancer cells failed to accumulate HIF-1α under hypoxic conditions, and thus tumor angiogenesis was blocked by *NRF2*-inhibition [29]. In a subsequent study, we identified that *miR-181c* elevation leads to the reductions in mitochondrial O_2_ consumption rate and ATP production in *NRF2*-silenced cancer cells by inhibiting mitochondrial function. As a molecular event, *miR-181c* directly represses the level of the mitochondria-encoded cytochrome c oxidase (MT-CO1), a catalytic subunit of the mitochondrial complex IV [30]. On the basis of these results, we hypothesized that *miR-181c* elevation might be a molecular link between *NRF2*-silencing and HIF-1α dysregulation in cancer cells. To test this idea, we compared levels of hypoxia-induced HIF-1α accumulation in breast cancer cells following the silencing of *NRF2* or the overexpression of *miR-181c*. In addition, we examined the effect of *NRF2*-silencing and *miR-181c* on the changes in hypoxic metabolic pathways regulated by HIF-1α.

## 2 Materials and methods

### 2.1 Reagents

The HIF-1α antibody was purchased from BD Biosciences (Palo Alto, CA). HK2, PDK1, LDHA, GLUT1 and LC3B antibodies were purchased from Cell Signaling Technology, Inc. (Danvers, MA, USA). The MT-CO1 antibody was purchased from Abcam (Cambridge, UK). Voltage-dependent anion channel (VDAC1), BCL2 interacting protein 3 (BNIP3) and β-tubulin were purchased from Santa Cruz Biotechnology, Inc. (Dallas, TX, USA). Puromycin was purchased from Sigma-Aldrich Co. (Saint Louis, MO, USA). The SYBR green real-time polymerase chain reaction (PCR) master mix was purchased from Takara Bio Inc. (Kusatsu, Shiga, Japan).

### 2.2 Cell culture

The MCF-7 and MDA-MB-231 human breast cancer cell lines were purchased from the American Type Culture Collection (ATCC, Manassas, VA, USA). Both cell lines were maintained in Dulbecco's Modification of Eagle's Medium (DMEM, Corning Life Sciences, Tewksbury, MA, USA) supplemented with 10% fetal bovine serum (FBS, Corning Life Sciences) and penicillin/streptomycin (Welgene, Inc., Daegu, Republic of Korea). The cells were grown at 37 °C in a humidified 5% CO_2_ atmosphere. The hypoxia incubation was performed in a hypoxia chamber water jacket incubator (Astec Co., Kasuya, Fukuoka, Japan) humidified with 1% O_2_ and 5% CO_2_ at 37 °C.

### 2.3 Establishment of *NRF2*-knockdown cells and miR181c overexpression cells

Lentiviral particles with shRNA were produced in HEK 293T as previously described [31]. HEK 293T cells were transfected with 1.5 μg pLKO.1-*NRF2* shRNA and the Mission Lentiviral Packaging Mix (Sigma-Aldrich, Co.) using Lipofectamine 2000 (Invitrogen Life technologies, Darmstadt, Germany). The pLKO.1-scrambled RNA (scRNA) plasmid was used as a nonspecific control RNA. On the second day, the medium with transfection complex was removed and each well was changed with the complete medium. Medium containing lentiviral particles was harvested after 4 days and used for subsequent transduction. MCF-7 and MDA-MB-231 cells were transduced with lentiviral particles containing either nonspecific scRNA or *NRF2* shRNA expression plasmids. Transduction was maintained for 48 h and followed by 24 h recovery in the complete medium. For the selection of cells with target plasmids, cells were grown in a medium containing under 1 μg/mL puromycin (Sigma-Aldrich Co.), as previously described [30]. The established *NRF2* shRNA-expressing cell lines were defined as shNRF2-MCF7 and shNRF2-MDA-MB-231, while the corresponding scrambled control cell lines were defined as scMCF7 and scMDA-MB-231. MCF-7 and MDA-MB-231 cells were stably transfected with pcDNA3-miR-181c plasmid to establish the miR-181c overexpression cell lines.

### 2.4 Isolation of microRNA (miRNA) and quantification by polymerase chain reaction (PCR) analysis

The miRNA was isolated from the cells with Trizol reagent (Ambion, Inc. Austin, TX, USA) according to the manufacturer's protocol. After the isolation, cDNA was synthesized with a miScript RT kit (Qiagen, Hilden, Germany) at 37 °C for 60 min followed by inactivation at 95 °C for 5 min. PCR analyses were performed with a miScript SYBR green PCR kit (Qiagen) using miRNA PCR forward primer of miR-181c (5′-AACATTCAA CCTGTCGGTGAGT-3′). Forward primer of U6 (5′-CGCAAGGATGACACGCAAATTC-3′) and RNU43 (5′-CTTATTGACGGGCGGACAGA-3′) were used as reference genes. All the primers were synthesized by Bioneer Corporation (Daejeon, Republic of Korea) as previously described [30]. The universal primer, which was provided in the miScript SYBR green PCR kit, was used as the reverse primer [30]. The reaction was carried out on LC480 LightCycler (Roche Diagnostics Deutschland GmbH, Mannheim, Germany) with initial denaturation at 95 °C for 15 min, 45 cycles of 95 °C for 15 s, 60 °C for 30 s, and 72 °C for 30 s. PCR analysis was carried out according to the Quantitative Real-Time PCR Experiments (MIQE) guidelines [32] as described below.

### 2.5 Isolation of total RNA and real-time PCR analysis

Sample preparation and RT-PCR analysis were performed according to the MIQE guidelines [32]. The total RNA was isolated from the cells using Trizol (Ambion) as described in the protocols [33]. A total of 200 ng RNA was transcribed into cDNA using GoScript RT (Promega) at 42 °C for 30 min followed by inactivation at 95 °C for 5 min, while no-RT sample was used as a negative control. The PCR was carried out using SYBR Green PCR MasterMix with primer of the human *HIF-1α, NRF2, NQO1, GCLC, GCLM* and *AKR1C1* as previously described [33] and glucose transporter 1 (*GLUT1,* NM_006516), hexokinase 2 (*HK2,* NM_000189), pyruvate dehydrogenase kinase (*PDK1,* NM_002610), lactate dehydrogenase kinase A (*LDHA,* NM_005566), Bcl2-interacting protein 3 (BNIP3, NM_004052) (Table 1). The reaction was carried out according to TB Green™ Premix Ex Taq™ II (Tli RNaseH Plus) protocols for LightCycler 480 System (Roche Diagnostics Deutschland GmbH, Mannheim, Germany). A total of 10 μL TB Green Premix Ex *Taq*II (Tli Rnase Plus) 2X, 20 pmoles forward and reverse primers, and 2 μL cDNA template were adjusted by nuclease free water to total reaction volume 20 μL. The PCR was performed with initial denaturation at 95 °C for 1 min, 45 cycles of 95 °C for 15 s, 60 °C for 30 s, and 72 °C for 40 s. Hypoxanthine guanine phosphoribosyl transferase (*HPRT,* NM_000194) and beta-2-microglobulin (*B2M*, NM_004048) primers were used as reference genes. All primers except *B2M* primer (Cosmogenetech, Seoul, Republic of Korea) were synthesized by Bioneer Corporation. The PCR efficiency calculated from slops of the calibration curves were: 99.25%, HPRT; 98.64%, B2M; 107.18%, GLUT1; 92.39%, LDHA; 95.18%, PDK1; 97%, HK2; 92.71%, BNIP3), and the *r*^*2*^ values of the calibration curves were over 0.99. Three independent experiments were performed with no-RT and no template control amplifications to examine DNA contamination, unspecific amplification, and primer dimerization. As the RNA integrity check, primers amplifying 5′ and 3′ regions of *HPRT* gene (Table 1) were used to assess the RNA quality in every sample. The samples with ratio of *HPRT* 5'/3′ between 0.9 and 1.1 were further processed for target gene quantification. As contamination assessment, we performed PCR analysis with no-RT controls (without RT enzyme) to evaluate the contamination of DNA in RNA samples. The assessment was achieved when no-RT controls and no template controls showed no amplification.

**Table 1:** Primer sequences for real-time PCR analyses.

Gene name	Forward	Reverse
*GLUT1*	5′-ATTGGCTCCGGTATCGTCAA-3′	5′-ATGGCCACGATGCTCAGATA-3′
HK2	5′-AGGATACGAGAAAACCGTGGG-3′	5′-GACACATCACATTTCGGAGCC-3′
*PDK1*	5′-TCCTGGACTTCGGATCAGTG-3′	5′-TGAACGGATGGTGTCCTGAG-3′
*LDHA*	5′-AGATTCCAGTGTGCCTGTATGG-3′	5′-AGAGAGTCCAATAGCCCAGGAT-3′
*BNIP3*	5′-GGTCAAGTCGGCCGGAAAAT-3′	5′-GACGCCTTCCAATATAGATCCCCA-3′
*HPRT*	5′-TGGCGTCGTGATTAGTGATG-3′	5′-GCTACA ATGTGATGGCCTCC-3′
*B2M*	5′-CTCACGTCATCCAGCAGAGA-3′	5′-CGGCAGGCATACTCATCTTT-3′
*HPRT 5′*	5′-CCTGGCGTCGTGATTAGTGA-3′	5′-GCTACAATGTGATGGCCTCC-3′
*HPRT3′*	5′-TGACACTGGCAAAACAATGC-3′	5′-CAAATCCAACAAAGTCTGGC-3′

### 2.6 Western blotting

The cells were washed using phosphate-buffered saline (PBS) supplemented with 100 μM MG132 and lysed with lysis buffer (50 mM Tris pH 7.4, 150 mM NaCl, 1 mM ethylene diamine tetra-acetic acid, 1% nonyl phenoxypolyethoxylethanol-40, 1% sodium dodecyl sulfate, and 10% sodium deoxycholate) containing a protease inhibitor cocktail (Sigma-Aldrich Co.). The protein concentrations were determined with bicinchoninic acid kit (Thermo Fisher Inc.,Waltham, MCA, USA). Proteins (15 mg/mL) were separated on 6%, 12% or 15% SDS-polyacrylamide gels, then transferred into nitrocellulose membranes (GE Healthcare Bio-Sciences, Pittsburgh, PA, USA). Membranes were blocked with 3% bovine serum albumin for 1 h and incubated overnight with the corresponding primary antibodies. Then, membranes were incubated with the secondary antibody combined with horseradish peroxidase (Thermo Fisher Inc.) for an hour. The chemoluminescent images were captured with a luminescent image analyzer ImageQuant LAS 4000 Mini (GE Healthcare Bio-Sciences) [34].

### 2.7 Mitochondria isolation

The mitochondria were isolated using a mitochondria isolation kit for cultured cells (Thermo Fisher Inc.) according to the manufacturer's instructions. Briefly, the mitochondria isolation reagent A was added to the cell pellets, and they were incubated on ice for 2 min. After that, mitochondria isolation reagent B was added to the cell suspension and mixed for 5 min at 4 °C with a vortex mixer. After the addition of the mitochondrial isolation reagent C, the lysates were centrifuged at 700×*g* for 10 min. The obtained supernatant was centrifuged at 12,000×*g* for 15 min. The mitochondria-containing pellets were obtained and washed with the mitochondrial isolation reagent C [30].

### 2.8 Measurement of extracellular lactate level

The extracellular lactate was measured using a Lactate Colorimetric/Fluorometric Assay Kit (BioVision, CA, USA) following manufacturer's instructions. Complete medium containing FBS was deproteinized through 10 kDa molecular weight spin filter (BioVision, CA, USA). The filtered samples were added 2–50 μl to a 96-well plate and adjust the volume to 50 μl/well with Lactate Assay Buffer. After that, Lactate Enzyme Mix (2 μl), Probe (2 μl) and Lactate Assay Buffer (46 μl) were added to adjusted samples, and they were incubated on room temperature for 30 min, protected from light. The optical density (OD) was detected at 570 nm using SPECTRO star^nano^ (BMG LABTECH GmbH, Ortenberg, Germany). The lactate levels were normalized to total cell number.

### 2.9 Cell number counting

The cells were seeded at a density of 3 × 10^5^ cells in 6 well plates. After incubation in hypoxia for 24 h, the cells were harvested. The cells were stained with trypan blue and the viable cells were calculated with a TC10 Automated Cell Counter (Bio-Rad Laboratories, Inc., Hercules, CA) as previously described [30].

### 2.10 Cell transfection

The cells were seeded in antibiotics-free complete medium at a density of 5 × 10^5^ cells and grown overnight. The next day, cells were transfected with either *BNIP3-* or *HIF-1α*-specific siRNA using Lipofectamine 2000 reagent (Life Technologies). After 24 h, the transfection complex-containing medium was removed, and the cells were incubated in complete medium for 2 h followed by hypoxia incubation. Cells were transfected with miR-181c inhibitor or negative control nucleotide as described previously [35].

### 2.11 Capillary Electrophoresis Time-of-Flight Mass Spectrometry (CE-TOFMS)-based metabolome analysis

The shNRF2- and sc-MCF7 cells (2–3 × 10^6^ cells) were incubated under hypoxic condition for 18 h and targeted metabolome analysis was performed using a Capillary Electrophoresis Time-of-Flight Mass Spectrometry (CE-TOFMS)-based analysis (Human Metabolome Technologies, Inc. (HMT Inc.), Mizukami, Japan) according to the provided instruction. Briefly, cells were washed with 5% (w/w) mannitol/Milli-Q water, and metabolites were extracted using methanol after the addition of internal standard solution (HMT Inc.). Then, extracted samples were filtered using a centrifugal filter units (HMT Inc.). Cationic and anionic metabolites were measured in the cation and anion mode, respectively using Agilent CE-TOFMS system (Santa Clara, USA) with a fused silica capillary (50 μm × 80 cm). Peak information (migration time, *m/z*, and peak area) was obtained using automatic integration software (MasterHands v. 2.17.11, Keio University, Japan). Then, putative metabolites were assigned from HMT's standard library based on determined *m/z* and migration time. At the same time, absolute quantification was carried out in target metabolites by normalizing peak area of each metabolite with respect to the area of the internal standard.

### 2.12 ^1^H-nuclear magnetic resonance (NMR)-based metabolite measurement

^1^H-NMR spectra were acquired on 600 MHz Agilent NMR spectrometer (Agilnet Technologies). For the analysis, 20 mg of sample and 20 μl of D_2_O with 2 mM TSP-d4 was added to NMR nano tube. For each sample, 128 transients were scanned. The spectra were Fourier transformed using Vnmrj (version 4.2 Agilent Technologies, Palo Alto, CA, USA) and all spectra were assigned and processed using Chenomx NMR Suite 7.1 professional and the Chenomx 600 MHz library database.

### 2.13 Measurement of autophagosome formation

The cells were detached by warm trypsin and washed using PBS. A total of 1 × 10^6^ cells were centrifuged at 1000 rpm for 5 min, suspended in assay buffer and stained using Cyto-ID Autophagy Detection Kit (Enzo, New York, USA). After incubation for 30 min, the cells were washed and autophagic vacuoles were determined using a 488 nm laser source in a Becton-Dickinson FACSCanto (Becton-Dickinson, SanJose, CA, USA) and FACSDiva software (Becton-Dickinson) as described previously [35].

### 2.14 Statistical analysis

The data were analyzed by Student's unpaired *t*-tests or two way analysis of variation (ANOVA) followed by the Benferroni's post test to determine significantly different group. Analyses was conducted with GraphPad Prism5 (GraphPad Software, Inc., La Jolla, CA, USA). Differences were considered significant at P < 0.05.

## 3 Results

### 3.1 HIF-1α dysregulation blocks activation of the glycolysis pathway in hypoxic *NRF2*-silenced breast cancer cells

**Fig. 1:**
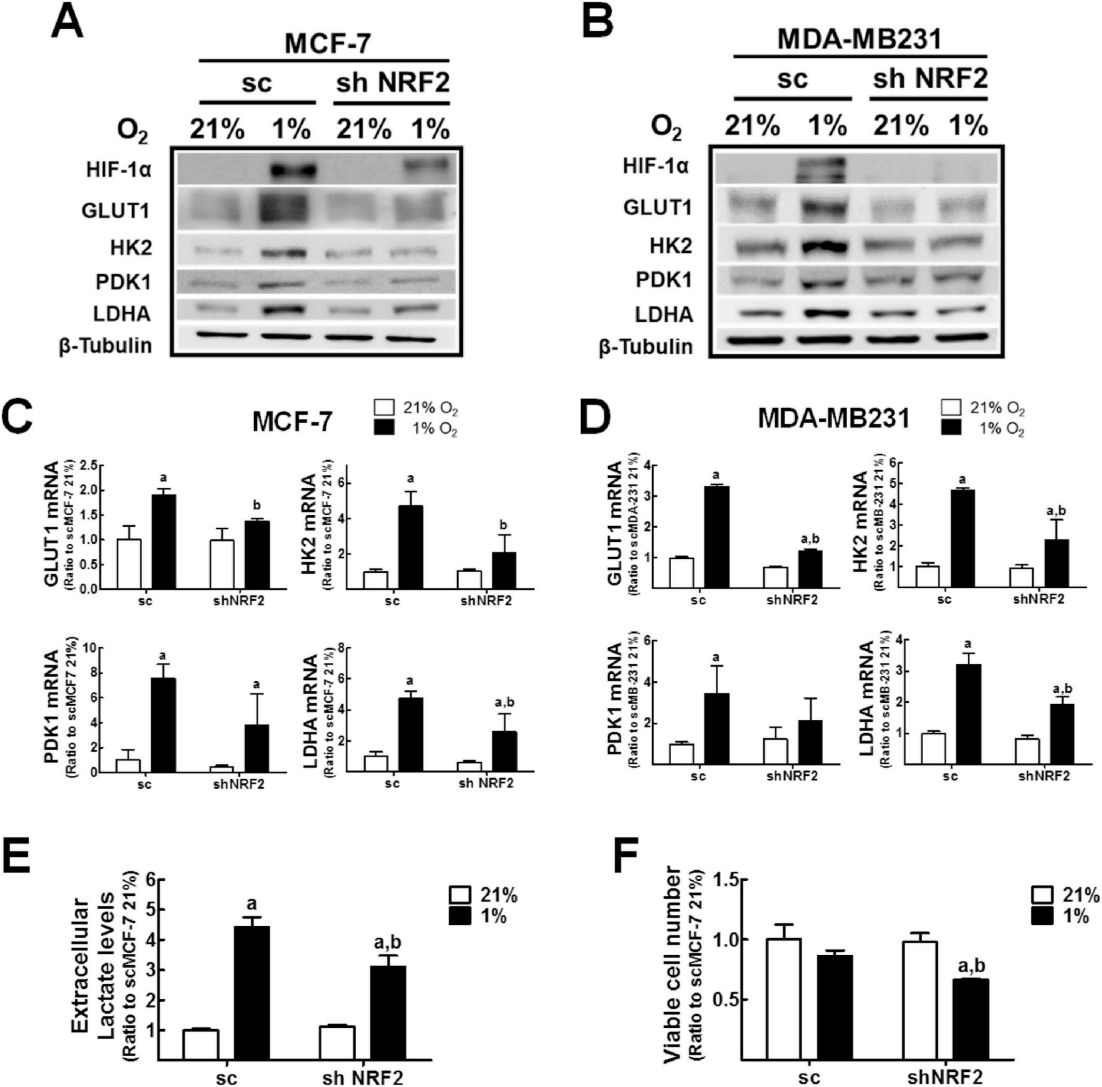
**Impaired HIF-1α and glycolysis-associated enzyme expression in hypoxic *NRF2*-knockdown breast cancer cells**. (A) The sc-MCF7 and shNRF2-MCF7 cells were incubated in normoxic (21% O_2_) and hypoxic (1% O_2_) condition for 24 h and protein levels of HIF-1α, GLUT1, HK2, PDK1, and LDHA were measured using western blotting. (B) Levels of HIF-1α, GLUT1, HK2, PDK1, and LDHA proteins were determined in sc-MDA-MB-231 and shNRF2-MDA-MB-231 cells. (C–D) Transcript levels of GLUT1, HK2, PDK1, and LDHA were assessed in NRF2-knockdown MCF7 (C) and MDA-MB-231 cells (D). HPRT and B2M were used for normalization of MCF7 levels (C), and HPRT was used for MDA-MB-231 normalization (D). (E) Extracellular lactate levels were measured in culture media from sc-MCF7 and shNRF2-MCF7 cells following hypoxic incubation for 18 h. (F) The sc-MCF7 and shNRF2-MCF7 cells were incubated in normoxic and hypoxic conditions for 24 h and cell viability was assessed using a cell counter. Data are presented as the means ± SD of three experiments. ^a^P <0.05 compared with each normoxic cell line. ^b^P < 0.05 compared with the hypoxic sc control group. Similar western blots were obtained in three independent experiments.

As an experimental system, we established a *NRF2*-knockdown MCF7 (shNRF2-MCF7) and MDA-MB-231 (shNRF2-MDA-MB-231) breast cancer cell lines and corresponding control cell lines (sc-MCF-7, sc-MDA-MB-231) [30], and *NRF2* knockdown has been confirmed (Supplementary Figs. S1A and 1B). MCF-7 and MDA-MB-231 breast cancer cell lines were shown to display lower levels of mitochondrial membrane potential (MMP) and ATP production following *NRF2* silencing [30], which are consistent phenomena to colon cancer cell lines, and therefore, these cell lines were chosen to ask whether *NRF2* knockdown-mediated HIF-1α dysregulation is a general phenomenon throughout cell lines. When these cells were incubated in a 1% O_2_ hypoxic condition for 24 h, levels of HIF-1α accumulation were substantially diminished in *NRF2*-silenced breast cancer cells compared to that in the control cell lines (Fig. 1A and B), which is in agreement with our previous report showing that HIF-1α accumulation is abrogated in *NRF2*-silenced colon cancer cells [29]. In line with the reduced HIF-1α levels, hypoxic *NRF2*-silenced breast cancer cells showed lower protein and mRNA levels of GLUT1 and glycolysis pathway-associated enzymes such as HK2, PDK1, and LDHA than hypoxic control cells (Fig. 1A−D). Notably, levels of extracellular lactic acid, which is a LDHA-mediated metabolic product of pyruvate, were significantly lower in *NRF2*-silenced MCF-7 compared to those in the control cells (Fig. 1E). Additionally, as a coherent result of suppressed glycolysis activation under hypoxic conditions, *NRF2*-knockdown MCF-7 cells showed a low viability following 24 h of hypoxic incubation (Fig. 1F). These results indicate that *NRF2*-silencing inhibits the glycolysis pathway activation under hypoxia via HIF-1α dysregulation.

### 3.2 Suppression of HIF-1α-inducible glycolysis in hypoxic *NRF2*-silenced cancer cells is mediated by *miR-181c*

**Fig. 2:**
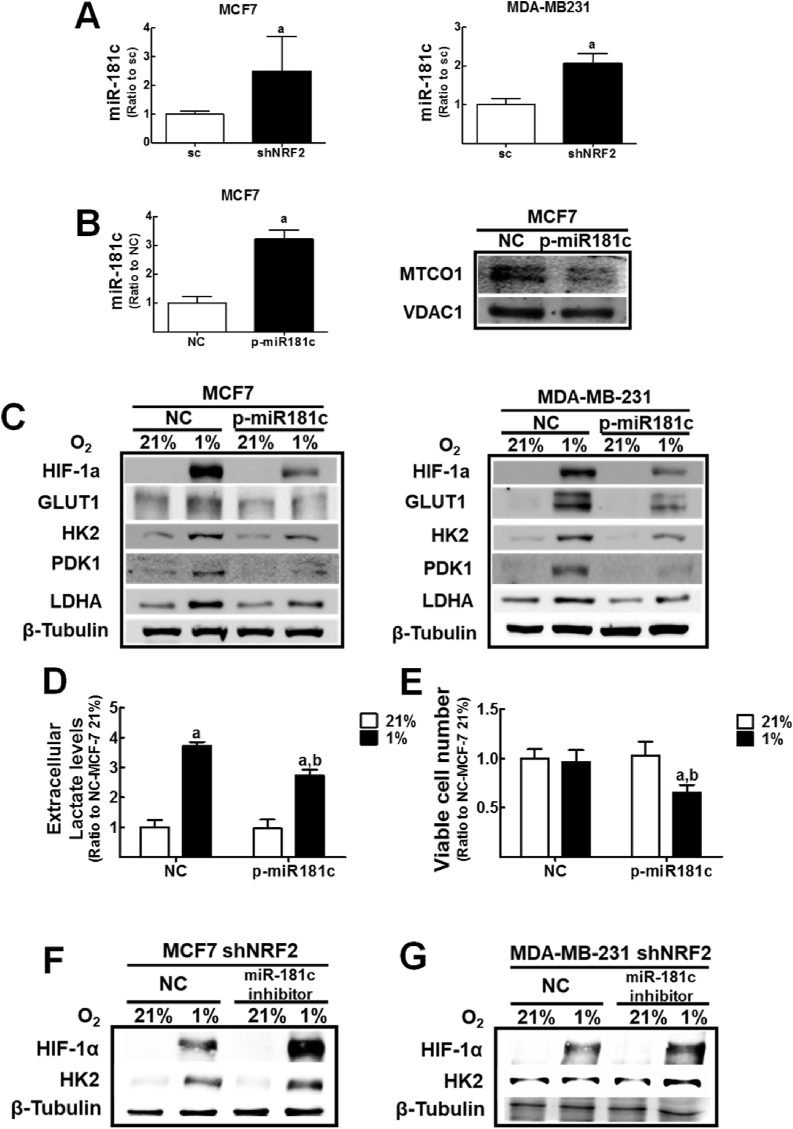
**Suppression of HIF-1α accumulation and glycolysis-associated enzyme expression by *miR-181c* overexpression**. (A) Levels of *miR-181c* were determined in *NRF2*-knockdown MCF7 and MDA-MB-231 cells. Levels of U6 and RNU43 were used for normalization of miR-181c in MCF7. MiR-181c levels in MDA-MB-231 were normalized using U6 levels. Data are presented as the means ± SD of three experiments ^a^P <0.05 compared to the sc control cells. (B) Levels of *miR-181c* and MT-CO1 were measured in *miR-181c* overexpressing MCF7 (p-miR181c) and corresponding empty plasmid-transfected MCF7 (NC) cells. MT-CO1 levels were determined in mitochondrial fractions. Data are presented as the means ± SD of three experiments ^a^P <0.05 compared to the empty plasmid control NC. VDAC was measured as a mitochondrial control marker. (C) Protein levels of HIF-1α, GLUT1, HK2, PDK1, and LDHA were assessed in p-miR181cMCF7 and MDA-MB-231 cell lines following hypoxic incubation for 24 h. (D) Extracellular lactate levels were measured in culture media from p-miR181c MCF7 and NC-MCF7 following hypoxia for 24 h. (E) The *miR-181c* overexpressing MCF7 and control cells were incubated under hypoxic conditions for 24 h and cell viability was determined using a cell counter. Data are presented as the means ± SD of three experiments. ^a^P <0.05 compared with each normoxic cell line. ^b^P < 0.05 compared with the hypoxic NC control group. (F–G) The *NRF2*-silenced (shNRF2)-MCF7 (F) or shNRF2-MDA-MB-231 cells (G) were transfected with a *miR-181c* inhibitor nucleotide (*miR-181c* inhibitor) or negative control nucleotide (NC) and HIF-1α and HK2 levels were assessed following hypoxic incubation for 24 h. Similar western blots were obtained in three independent experiments.

Based on our previous observation of the role of *miR-181c-5p* (*miR-181c*) in MT-CO1 suppression and consequent reduction in MMP and ATP production in *NRF2*-knockdown colon cancer cells [30], we speculated that *miR-181c* is involved in HIF-1α dysregulation in *NRF2*-silenced breast cancer cells. First, similar to colon cancer cells, *miR-181c* levels were elevated by *NRF2*-silencing in both breast cancer cell lines (Fig. 2A). Second, in order to examine the direct link between *miR-181c* and HIF-1α dysregulation, *miR-181c* was stably overexpressed in both breast cancer cell lines. The overexpression of *miR-181c* reduced MT-CO1 levels in both breast cancer cell lines (Fig. 2B and Supplementary Fig. S2). Additionally, HIF-1α accumulation and hypoxia-induced levels of glycolysis-associated proteins such as GLUT1, HK2, PDK1, and LDHA were significantly diminished in hypoxic *miR-181c* MCF-7 and MDA-MB-231 when compared to the cell lines in normoxia (Fig. 2C). As a result of repressed glycolysis metabolism, *miR-181c* overexpression suppressed hypoxic-inducible lactate production, which is similar to that in *NRF2*-silenced cells (Fig. 2D), and diminished cell viability under hypoxic incubation (Fig. 2E). Notably, the introduction of a *miR-181c* inhibitor in *NRF2*-silenced breast cancer cells restored HIF-1α accumulation and HK2 elevation following hypoxic incubation (Fig. 2F and G). These results clearly show that *miR-181c* elevation is a molecular mediator for the dysregulation HIF-1α signaling in *NRF2*-silenced breast cancer cells.

### 3.3 Metabolome profiling shows that hypoxia-inducible glycolysis and PPP metabolites are suppressed by *NRF2*-silencing

**Fig. 3:**
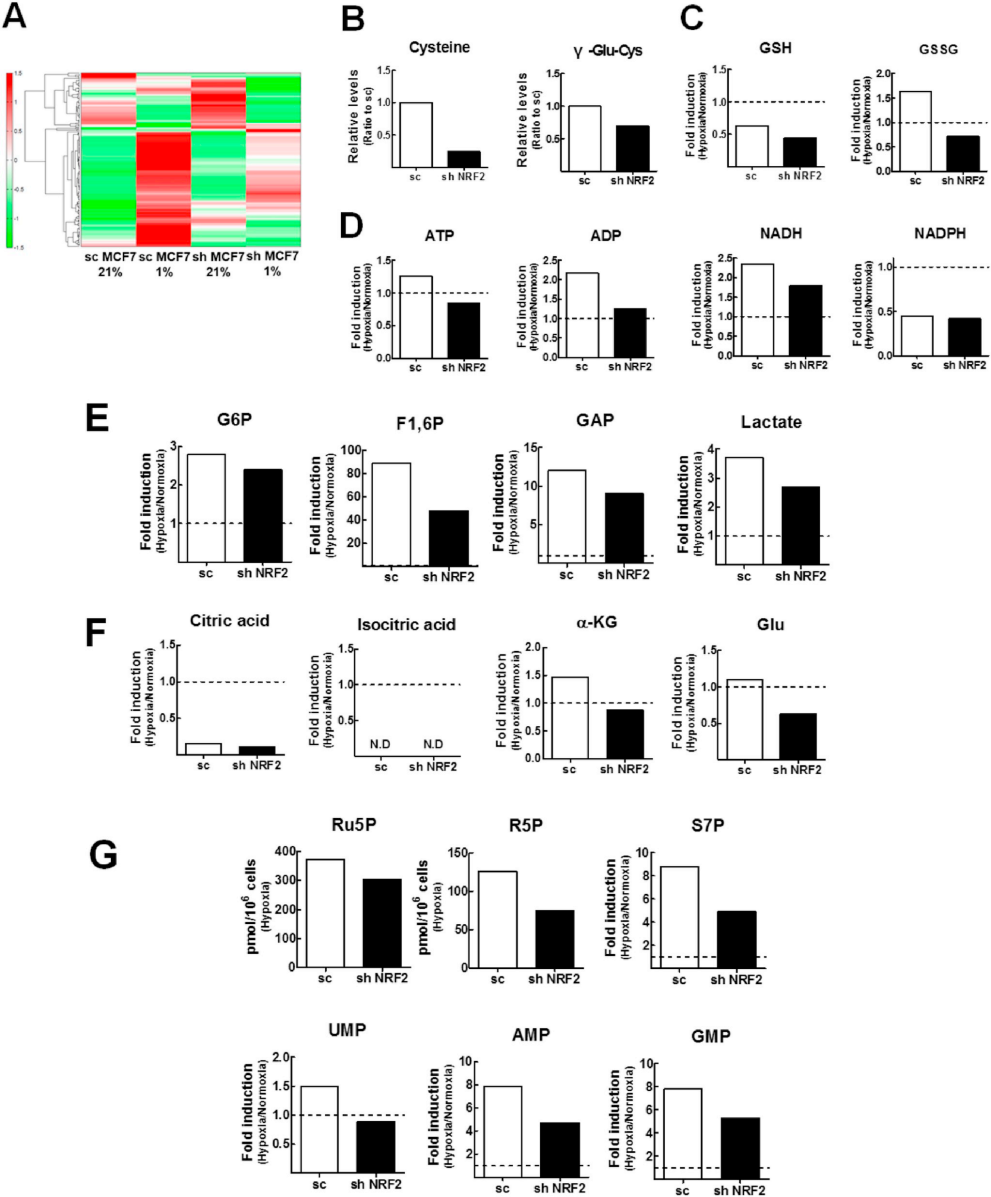
**Metabolome profiles of *NRF2*-knockdown MCF7 cells following hypoxic incubation**. (A) Global metabolomes were obtained in normoxic and hypoxic *NRF2*-silenced MCF7 cells based on CE-TOFMS analysis. The heatmap of quantified global metabolites in normoxic (21% O_2_, 18 h) and hypoxic (1% O_2_, 18 h) cells is presented. (B) Levels of Cys and γ-Glu-Cys in the sc-MCF7 and shNRF2-MCF7 cells. (C) Relative levels of GSH and GSSG in hypoxic sc-MCF7 and shNRF2-MCF7 with respect to each normoxic level. (D) Relative levels of ATP, ADP, NADH, and NADPH in hypoxic sc-MCF7 and shNRF2-MCF7 with respect to each normoxic level. (E) Relative levels of glycolysis metabolites glucose-6-phosphate (G6P), fructose-1,6-diphosphate (F1,6P), glyceraldehyde-3-phosphate (GAP), and lactate in hypoxic sc-MCF7 and shNRF2-MCF7 with respect to each normoxic level. (F) Relative levels of TCA metabolites such as citric acid, isocitric acid, α-ketoglutarate (α-KG), and glutamate (Glu) hypoxic sc-MCF7 and shNRF2-MCF7 with respect to each normoxic level. (G) Quantified amount of ribulose-5-phosphate (Ru5P) and ribose-5-phosphate (R5P) in hypoxic sc-MCF7 and shNRF2-MCF7 cells. Relative levels of sedoheptulose-7-phosphate (S7P), UMP, AMP, and GMP in hypoxic sc-MCF7 and shNRF2-MCF7 with respect to each normoxic level.

To examine the effect of *NRF2*-silencing on HIF-1α-mediated metabolic changes in hypoxic breast cancer cells, global metabolome profile was analyzed in cell extracts from hypoxic *NRF2*-silenced and control MCF7 cells using the CE-TOFMS method. A total of 216 metabolites, including carbohydrates, amino acids, nucleotides and organic acids were identified with this method (Fig. 3A). First, *NRF2*-knockdown reduced the basal levels of glutathione (GSH) precursors cysteine (Cys) and γ-Glu-Cys compared to the control MCF-7 cells, which confirms the NRF2-dependent import of Cys into the cell (Fig. 3B). When both cell lines were incubated under hypoxic conditions for 24 h, GSH levels in both cell lines were repressed and the level of GSSG, the oxidized form of GSH, was higher in the hypoxic control MCF-7 cells as compared with hypoxic *NRF2*-knockdown cells (Fig. 3C). Second, levels of ATP, ADP, and NADH were slightly elevated in hypoxic control cells, whereas those levels were low in *NRF2*-silenced cell as compared with the control cells (Fig. 3D). NADPH levels were suppressed following hypoxia in both cell lines (Fig. 3D). Third, in line with differential elevations in HIF-1α and glycolysis pathway enzymes, levels of hypoxia-inducible glycolysis metabolites, including G6P, fructose 1.6-diphosphate (F1,6P), and glyceraldehyde 3-phosphate (GAP) were lower in *NRF2*-silenced cells as compared with the control cells (Fig. 3E). As a result of this, the cellular level of lactate, which was inducible by hypoxia, was diminished by *NRF2*-silencing (Fig. 3E). Fourth, levels of TCA cycle-associated metabolites such as citric acid and isocitric acid were substantially reduced in both cell lines under hypoxia compared with each in normoxia, indicating the suppression of TCA cycle resulting from the elevation in PDK1 in the absence of O_2_ (Fig. 3F). The level of α-ketoglutarate (α-KG), the next TCA metabolite, was recovered under hypoxia, which implies that there is metabolic flux from glutamate pathway. Notably, the levels of α-KG and glutamate were reduced by *NRF2*-silencing (Fig. 3F). Fifth, in addition to being the primary source of energy in hypoxic cells, glucose and glycolysis intermediates act as substrates for the synthesis of cellular macromolecules. Hypoxia substantially elevated PPP metabolites such as ribulose-5-phosphate (Ru5P), ribose-5-phosphate (R5P), and sedoheptulose-7-phosphate (S7P) in both cell lines, and these elevations were lower in *NRF2*-silenced MCF-7 (Fig. 3G). Similarly, hypoxia-mediated elevations in nucleotide levels (UMP, AMP, GMP) were likewise lower with *NRF2*-knockdown (Fig. 3G), indicating that the PPP-derived nucleotide synthesis pathway is diminished in hypoxic *NRF2*-silenced cells. These results demonstrate that hypoxic breast cancer cells undergo metabolic changes to survive, which involves facilitating the glycolysis pathway, suppressing TCA cycle, which consequently elevates lactate production and activates PPP-mediated nucleotide synthesis. All of these metabolic changes are relatively impaired in *NRF2*-knockdown breast cancer cells, presumably as a result of HIF-1α dysregulation.

### 3.4 Metabolome analysis reveals that hypoxia-mediated catabolism is suppressed by *NRF2*-silencing

**Fig. 4:**
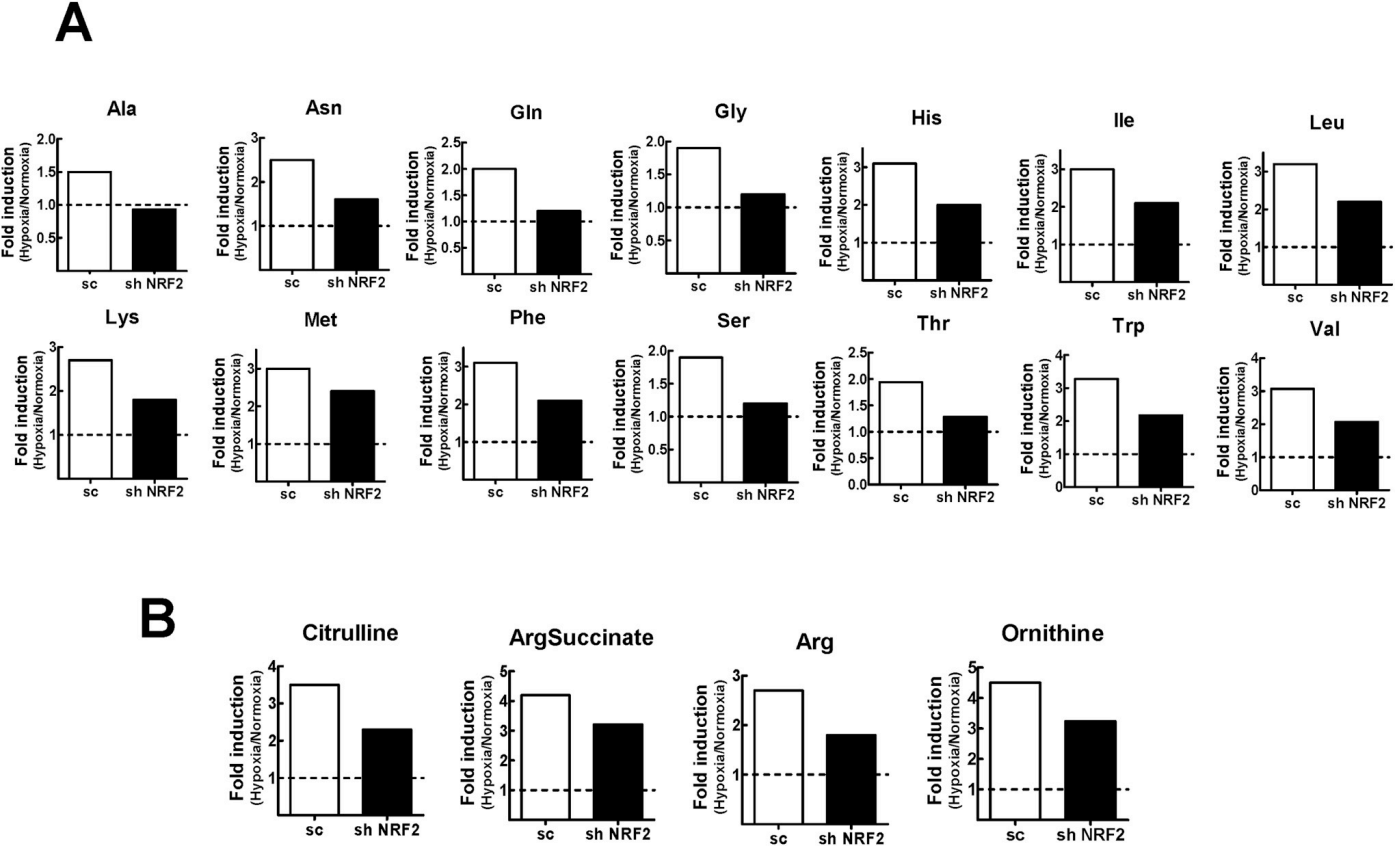
**Diminished hypoxia-inducible elevations in amino acids and urea cycle metabolites in *NRF2*-knockdown MCF7**. (A) Relative levels of amino acids (Ala, Asn, Gln, Gly, His, Ile, Leu, Lys, Met, Phem Ser, Thr, Trp, and Val) in hypoxic sc-MCF7 and shNRF2-MCF7 with respect to each normoxic level. (B) Relative levels of urea cycles metabolites such as citrulline, argininosuccinic acid (ArgSuccinate), arginine (Arg), and ornithine in hypoxic sc-MCF7 and shNRF2-MCF7 with respect to each normoxic level.

In addition to the changes in glycolysis, TCA, and PPP metabolites, the changes in amino acids levels were notable in hypoxic cancer cells. The levels of most amino acids were increased by 2−3-fold following hypoxic incubation and, interestingly, these elevations were suppressed by *NRF2*-silencing (Fig. 4A). In line with this, the levels of metabolites (citrulline, arg-succinate, arginine, and ornithine) of the urea cycle, a biochemical reaction converting amino acid-derived ammonia (NH_3_) to urea, were likewise increased by hypoxic incubation and these increased levels were lower in *NRF2*-silenced cells than in the control cells (Fig. 4B). These results indicate that hypoxic cancer cells might stimulate catabolic processes to compensate for the stress which occurs under hypoxic conditions.

### 3.5 HIF-1α-mediated autophagy activation is suppressed in hypoxic *NRF2*-silenced cells

**Fig. 5:**
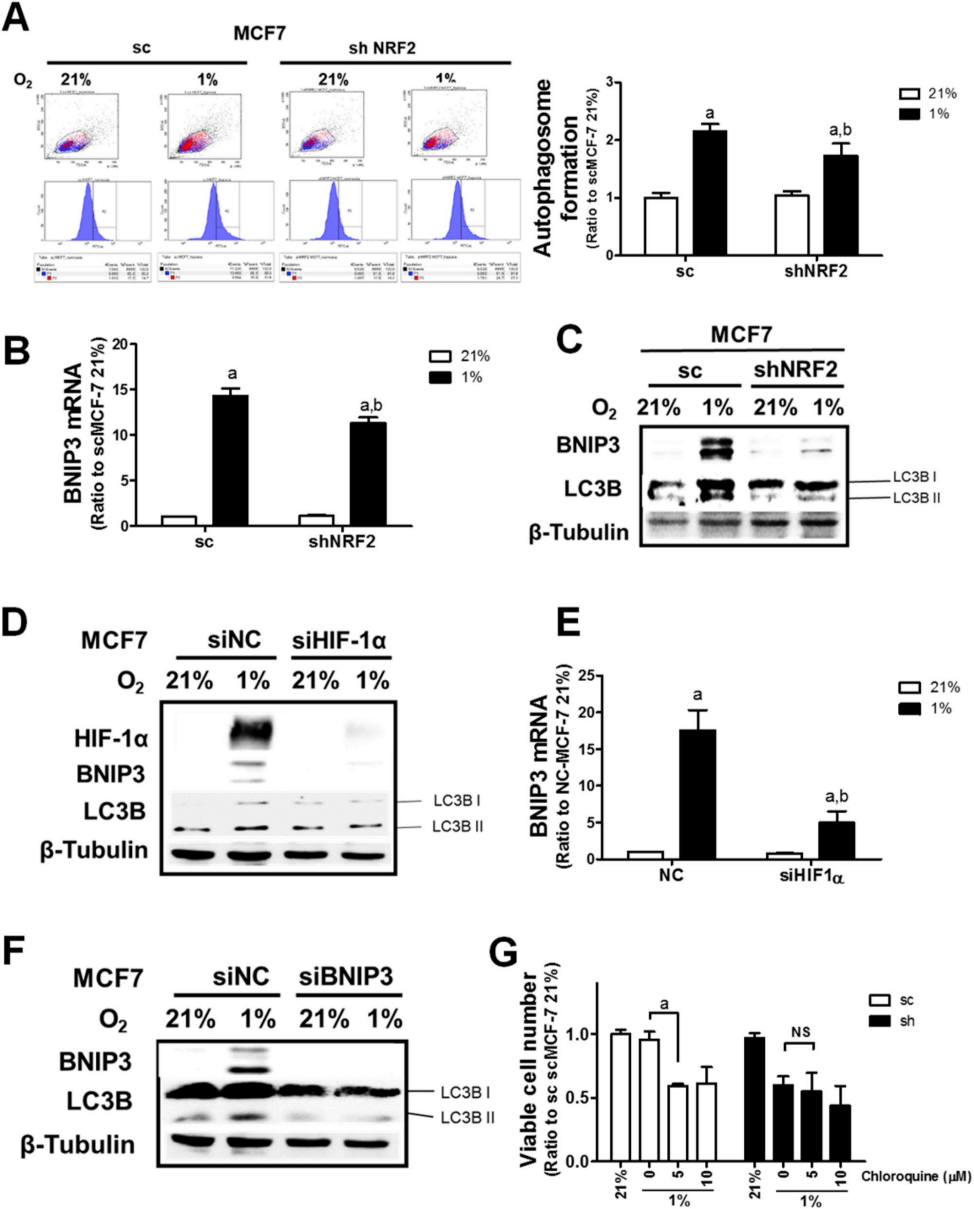
**Suppression of hypoxia-inducible autophagy activation by *NRF2*-silencing**. (A) Autophagic vacuole formation was monitored in sc-MCF7 and shNRF2-MCF7 cells following hypoxic incubation for 24 h. The bar graph represents relative autophagosome formation levels from three experiments. Data are presented as the means ± SD. ^a^P <0.05 compared with each normoxic cell line. ^b^P < 0.05 compared with the hypoxic sc control group. (B) Transcript levels for *BNIP3* in sc-MCF7 and shNRF2-MCF7 following hypoxic incubation for 24 h. HPRT and B2M were used as reference genes for normalization. Data are presented as the means ± SD from three experiments. ^a^P <0.05 compared with each normoxic cell line. ^b^P < 0.05 compared with the hypoxic sc control group. (C) Protein levels of BNIP3 and LC3BII were determined following hypoxic incubation for 24 h. (D) The control MCF7 cells were transiently transfected with *HIF-1α*-specific siRNA (HIF-1α) or nonspecific siRNA (siNC) and protein levels of HIF-1α, BNIP3, and LC3BII were assessed using western blotting. (E) Levels of *BNIP3* mRNA were determined in hypoxic MCF7 cells following *HIF-1α* siRNA introduction. HPRT and B2M were used as reference genes for normalization. Data are presented as the means ± SD from three experiments. ^a^P <0.05 compared with each normoxic cell line. ^b^P < 0.05 compared with the hypoxic NC control group. (F) The control MCF7 cells were transiently transfected with *BNIP3*-specific siRNA (siBNIP3) or nonspecific siRNA (siNC) and protein levels of BNIP3 and LC3BII were monitored using western blotting. (G) The sc-MCF7 and shNRF2-MCF7 were incubated under hypoxia for 24 h in the presence of chloroquine (5 and 10 μM) and cell viability was assessed using a cell counter. Data are presented as the means ± SD from three experiments. ^a^P <0.05 compared with each chloroquine-untreated hypoxic cell line. NS, statistically not significant. Similar western blots were obtained in three independent experiments.

One of the major catabolic programs under hypoxic conditions is autophagy [20]. In order to examine autophagy activation, the level of autophagic vacuole (autophagosome) formation was monitored using flow cytometry. Hypoxic incubation for 24 h elevated the level of autophagic vacuoles, which is in line with the elevation in catabolic metabolites, and *NRF2*-knockdown repressed hypoxia-induced autophagy activation (Fig. 5A). BNIP3, a mitochondrial pro-apoptotic protein, has been known to activate the autophagy process under HIF-1α accumulation [36]. In hypoxic MCF-7 cells (24 h), the level of BNIP3 mRNA was elevated, and it was notable that BNIP3 elevation in *NRF2*-silenced cells was suppressed (Fig. 5B). Protein levels of BNIP3 and the autophagy-specific adaptor protein LC3BII were increased upon hypoxia, and these elevations were suppressed by *NRF2*-knockdown (Fig. 5C). Similar results were obtained in hypoxic MDA-MB-231 cells (Supplementary Figs. S3 and S4). In order to explore the role of HIF-1α and BNIP3 in autophagy activation, *HIF-1α* and *BNIP3* were silenced and the level of the autophagy marker LC3BII was determined in MCF-7 cells. First, *HIF-1α*-silencing attenuated the hypoxia-mediated elevations of BNIP3 and LC3BII compared to the control siRNA group (Fig. 5D). Transcript levels of *BNIP3* were significantly diminished by *HIF-1α*-silencing (Fig. 5E). Similar results were obtained from MDA-MB-231 cells (Supplementary Figs. S5 and S6). Second, *BNIP3*-silencing blocked hypoxia-mediated LC3BII elevation in MCF-7 cells (Fig. 5F), indicating that autophagy activation in hypoxic cells is BNIP3-dependent. These results suggest that *HIF-1α*-mediated BNIP3 induction led to autophagy activation in breast cancer cells and, thus, *NRF2*-silenced cells exhibited autophagy suppression as a result of HIF-1α/BNIP3 disruption. Higher levels of autophagy activation in the control MCF-7 cells might imply that there is an autophagy-dependent adaptive survival program in response to hypoxic conditions. Indeed, the incubation of the control MCF-7 cells with the autophagy inhibitor chloroquine decreased cell viability in hypoxic conditions, whereas the effect of chloroquine was minimal in *NRF2*-silenced MCF-7 cells under hypoxia (Fig. 5G).

### 3.6 Repressed autophagy activation in hypoxic *NRF2*-silenced cells is mediated by *miR-181c* elevation

**Fig. 6:**
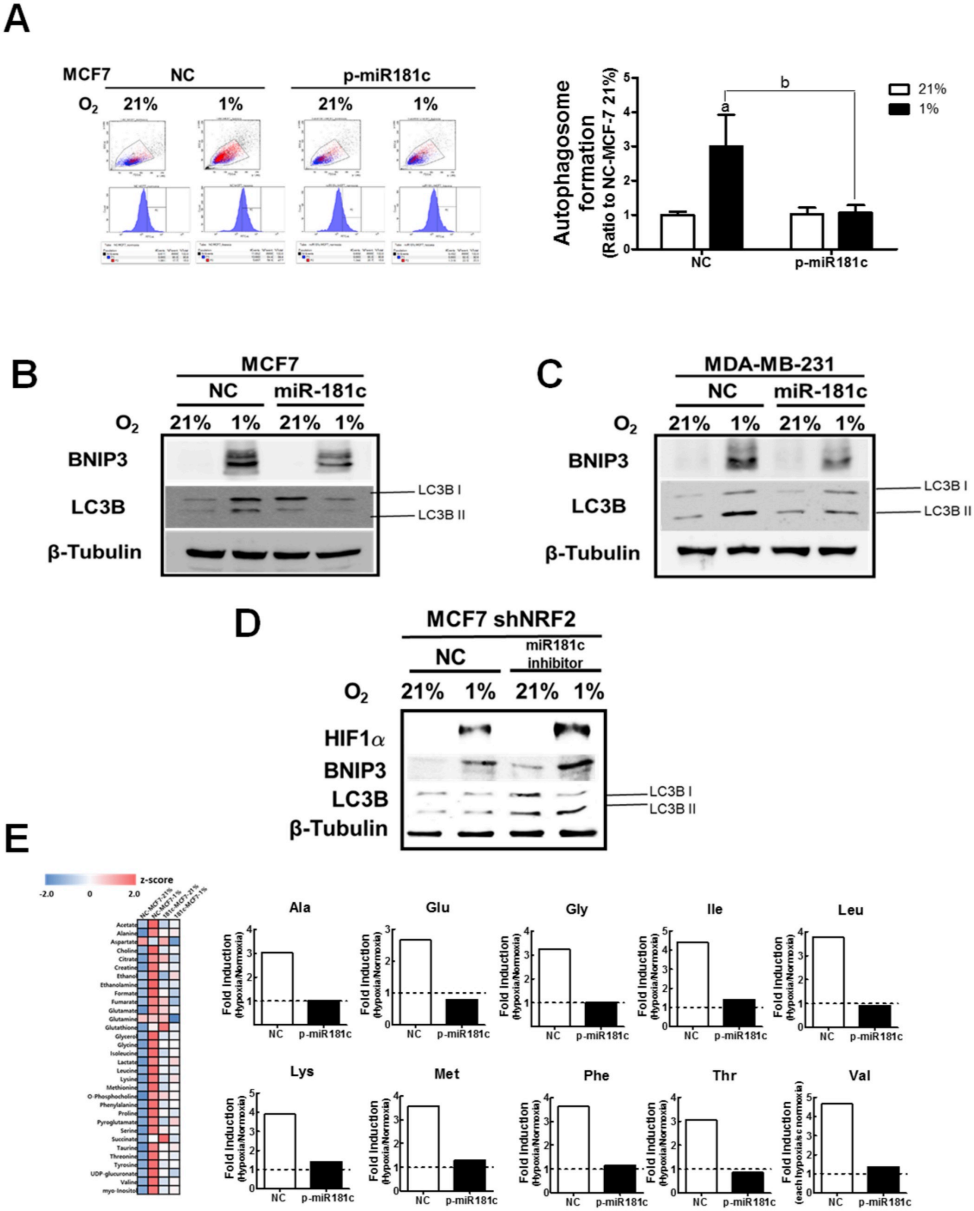
**Reduction in hypoxia-inducible autophagy activation by *miR-181c* expression**. (A) Autophagic vacuole formation was determined in MCF7 cells overexpressing *miR-181c* (p-miR-181c) and control cells (NC) following hypoxic incubation for 24 h. The bar graph represents relative autophagosome formation levels from three experiments. Data are presented as the means ± SD. ^a^P <0.05 compared with each normoxic cell line. ^b^P < 0.05 compared with the hypoxic NC control group. (B) Protein levels of BNIP3 and LC3BII were determined in MCF7 cells overexpressing *miR-181c* (p-miR-181c) and control cells (NC) following hypoxic incubation for 24 h. (C) Protein levels of BNIP3 and LC3BII were determined in MDA-MB-231 cells overexpressing *miR-181c* (p-miR-181c) and control cells (NC) following hypoxic incubation for 24 h. (D) The shNRF2-MCF7 cells were transiently transfected with a *miR-181c* inhibitor nucleotide (miR181c inhibitor) or a nonspecific nucleotide (NC) and protein levels of HIF-1α, BNIP3, and LC3BII were assessed using western blotting. (E) Target metabolites were analyzed in MCF7 cells overexpressing *miR-181c* (p-miR-181c) and control cells (NC) following hypoxic incubation for 24 h. Heatmap of quantified metabolites is presented. Relative levels of amino acids (Ala, Glu, Gly, Ile, Leu, Lys, Met, Phe, Thr, and Val) in hypoxic p-miR181c MCF7 are expressed as ratios with respect to each normoxic level.

In order to examine the involvement of *miR-181c* in autophagy inhibition in hypoxic *NRF2*-silenced cells, autophagy activity was assessed in MCF-7 cells overexpressing *miR-181c*. Similar to *NRF2*-knockdown breast cancer cells, hypoxia-induced autophagic vacuole formation was blunted by expressing *miR-181c* in MCF-7 cells (Fig. 6A). Hypoxia-mediated BNIP3 and LC3BII elevations were likewise repressed by expressing *miR-181c* in breast cancer cells (Fig. 6B and C). Additionally, autophagy repression in *NRF2-*knockdown cells could be reversed by a *miR-181c* inhibitor sequence (Fig. 6D), suggesting a critical role for *miR-181c* in autophagy inhibition in *NRF2*-silenced cells. Similarly, miR-181c inhibitor treatment restored BNIP3 and LC3BII following hypoxia (Supplementary Fig. S7). In line with these results, a ^1^H-NMR-based metabolome analysis revealed that the hypoxia-mediated increase in amino acids was blocked by overexpressing *miR-181c* in breast cancer cells (Fig. 6E). These results suggest that *miR-181c* elevation led to the impairment in HIF-1α/BNIP3-mediated autophagy activation in *NRF2*-silenced breast cancer cells.

## 4 Discussion

Because NRF2 and HIF-1α are critical factors for sensing O_2_ and its related ROS, the mechanism of how NRF2 and HIF-1α are co-regulated in hypoxic conditions is intriguing. Our current study shows that *NRF2*-silencing suppresses hypoxia-inducible HIF-1α accumulation in breast cancer cells, and thereby inhibits the HIF-1α-mediated metabolic adaptation, including glycolysis activation, PPP facilitation, and autophagy stimulation, which eventually impairs the viability of *NRF2*-silenced cancer cells in hypoxic environment. Notably, we demonstrate that the *NRF2*-silencing effect on HIF-1α is mediated by *miR-181c* elevation. Breast cancer cells with *miR-181c* overexpression exhibited similar behaviors upon hypoxia: HIF-1α accumulation was attenuated and the levels of glycolysis enzymes were suppressed. Moreover, the inhibitory effect of *NRF2*-silencing on HIF-1α was blocked by treatment with a *miR181c* inhibitor. These results suggest a strong correlation between NRF2 and HIF-1α in the adaptive regulation of metabolic pathways, and further imply beneficial effects of *NRF2*-silencing on HIF-1α-mediated metabolic adaptation under hypoxic tumor environment.

The role of *miR-181c* in mitochondria was first demonstrated in a study using cardiac cells from rats [37]. In this study, *miR-181c* was identified as a mitochondria-localized miRNA, and was shown to increase O_2_ consumption and MMP by targeting MT-CO1 of the electron transport chain complex IV. Our previous study identified *miR-181c* as one of the miRNAs induced following *NRF2*-silencing in both HT29 and HCT116 colon cancer cells [30]. However, unlike a study by Das et al. [37], we found that *miR-181c*-mediated electron transport chain dysfunction led to a decrease in MMP, mitochondrial respiration rate, and ATP production in normoxic cancer cells. As these changes could be partly compensated for by the activation of adenosine monophosphate (AMP)-activated protein kinase-a (AMPKα) signaling and consequent adaptive metabolic pathways to maintain energy homeostasis, *NRF2*-silenced cancer cells are vulnerable to AMPKα inhibition, which suggests the potential for combined inhibition of NRF2 and AMPKα to overcome adaptive behaviors of cancer cells [30]. In addition to the role in normoxic cancer metabolism, our current study elucidated a novel role of *miR-181c* in hypoxic breast cancer cells. *MiR-181c* suppressed HIF-1α stabilization under hypoxic conditions and blocked the HIF-1α-mediated adaptive metabolic changes in glycolysis and autophagy. In particular, the inhibitory effect of *miR-181c* on HIF-1α accumulation can be attributed to the O_2_ redistribution effect which has been well-described in nitric oxide (NO)-treated cells [38,39]. When mitochondrial respiration is repressed by NO under hypoxic conditions, O_2_ is redistributed to non-respiratory targets such as PHD, allowing PHD to retain its enzymatic activity for HIF-1α hydroxylation, resulting in the inhibition of HIF-1α signaling. Indeed, HIF-1α accumulation was recovered in anoxic (0.1% O_2_) *NRF2*-silenced cancer cells [29] and *miR-181c* overexpressing breast cancer cells (data not shown). Several recent studies have indicated a role of *miR-181c* as a tumor suppressor. *MiR-181c* inhibited metastasis and migration in glioblastoma [40] and its overexpression in myelocytic leukemia cells suppressed chemoresistance [41]. Reduced *miR-181c* expression has been associated with gastric cancer in patients [42]. Together with these reports, our study suggests the beneficial effects of *miR-181c* for the control of the HIF-1α-mediated adaptive cancer behaviors in the hypoxic tumor microenvironment.

Metabolic adaptation is an important survival strategy for cancer cells within the hypoxic tumor environment. Under the condition of O_2_ limitation, metabolic pathways shift from energy-efficient oxidative phosphorylation to the anaerobic glycolysis pathway for the purpose of ATP generation [13,43]. This change is achieved by the upregulation of the HIF-1α-mediated glycolysis-involved GLUT1, HK2 and PDK1, an inhibitor of the conversion of pyruvate to acetyl CoA [43]. At the same time, the formation of lactic acid from pyruvate is facilitated by the HIF-1α-mediated upregulation of LDHA, which contributes to the acidification of the tumor microenvironment [44]. In our results, all of these hypoxia-induced metabolic changes were suppressed by *NRF2*-silencing. The HIF-1α-inducible expressions of GLUT1, HK2, PDK1, and LDHA were low in *NRF2*-silenced breast cancer cells and, accordantly, levels of hypoxia-inducible glycolysis metabolites such as G6P, F16P, GAP, and lactate were diminished by *NRF2*-silencing. As a result of PDK1 elevation, levels of the TCA metabolites citric acid and isocitric acid were substantially reduced following hypoxia, which provides evidence that the TCA cycle is inhibited under hypoxic conditions. However, the elevation of α-KG levels in control cells might imply the contribution of the glutaminolysis pathway in the TCA cycle [28,45]. However, *NRF2*-silenced cells did not show α-KG elevation. In line with the inhibition of glycolysis pathway, *NRF2*-silenced MCF-7 cells exhibited lower levels of HIF-1α-induced ATP and ADP and cell viability under hypoxic conditions. Similar to *NRF2*-silenced MCF-7, overexpressing *miR-181c* caused suppression of hypoxia-induced glycolysis enzymes and cell viability.

In addition to the glycolysis pathway, activation of the PPP is another core feature of hypoxic cancer cells to provide necessary cellular building blocks [46,47]. The HIF-1α-mediated activation of the PPP provides precursors for nucleotide synthesis, which aids rapid proliferation of cancer cells under energy-limited hypoxic conditions. PPP activation additionally enhances NADPH levels and maintains redox homeostasis by providing reducing power to antioxidant enzymes such as GSH reductase [48]. Our metabolome analysis revealed that hypoxia-inducible levels of Ru5P, R5P, and S7P were low in *NRF2*-silenced breast cancer cells, indicating the suppression of the PPP. In agreement with this, hypoxic levels of PPP-derived nucleotide precursors were lower in *NRF2*-silenced breast cancer cells than in control cells. These results indicate that the supply of building blocks can be restricted by NRF2 inhibition in hypoxic cancer cells, which is supported by reduced cell viability following hypoxic incubation in *NRF2*-silenced cells. Levels of NADPH were similarly diminished under hypoxic conditions in both cell lines, implying the extensive consumption of NADPH in hypoxic conditions.

It is noteworthy that NRF2 can upregulate multiple metabolic genes, which can lead to metabolic shift in normoxic condition. Expression of PPP enzymes such as G6P dehydrogenase (G6PD), 6-phosphogluconate dehydrogenase (PGD), and transketolase (TKT) was directly regulated by NRF2 in lung cancer cells, and NRF2 overexpression redirected cell metabolism to PPP-mediated purine nucleotide synthesis [28]. In another study, NRF2-mediated regulation of PPP genes was demonstrated to be an indirect event: NRF2 signaling diminished the expression of *miR-1* and *miR-206*, which are inhibitor miRNAs for PPP enzymes such as G6PD and TKT and this, in turn, led to an elevation in PPP metabolism [49]. Additionally, lung adenocarcinoma with simultaneous mutations in *Keap1* and *Kras* showed an increased glutamine utilization for energy production [45]. NRF2 also facilitates the metabolism from glucose to serine by indirectly regulating its biosynthetic enzymes [50]. These results indicate that NRF2 is positively involved in facilitation of glucose metabolism toward PPP and serine synthesis, and enhancement of glutaminolysis, which all together promotes tumor proliferation in normoxic condition. Therefore, *NRF2* silencing inhibited PPP metabolism and consequently suppressed tumor cell proliferation by restricting purine nucleotides supply [45,49], which implies the beneficial effects of NRF2 inhibition in both normoxic and hypoxic tumor environments.

In hypoxic breast cancer cells, BNIP3-mediated autophagy activation and elevation of amino acid levels were notable. BNIP3, a BCL-2 family protein, is known to be transcriptionally regulated by HIF-1α and to be involved in cancer cell death in hypoxic conditions [51]. As a link to autophagy, hypoxia-induced Bnip3 was initially identified to be critical in autophagic cell death in murine embryonic fibroblasts (MEFs) [36]. Then, it was shown that BNIP3 is essential for hypoxia-induced autophagy in cancer cells and disruption of *BNIP3* triggered cell death, which suggests a positive role of HIF-1α, BNIP3, and autophagy in tumor survival and progression [52]. Of particular note, this study shows that the BH3 domain of BNIP3 disrupts the Bcl2-Beclin complex, which results in the stabilization of the BNIP3/Bcl2 complex and subsequently releases Beclin to activate the pro-survival autophagy pathway. Now, it is generally accepted that hypoxia-induced autophagy plays a crucial role in cancer cell survival by removing damaged cellular components and supplying a source of nutrients under conditions of hypoxia without nutrient deficits, whereas severe stress conditions with hypoxia and nutrient limitation causes autophagic cell death [53]. Our study shows that hypoxia-induced autophagy activation is mediated by HIF-1α/BNIP3 signaling and *NRF2*-silencing inhibited the HIF-1α-mediated autophagy activation and amino acid elevation. Similarly to HIF-1α-mediated metabolic changes, *miR-181c* was found to be involved in autophagy activation; *miR-181c* expression suppressed BNIP3-mediated autophagy activation and hypoxia-inducible amino acid elevation. Additionally, the inhibition of *miR-181c* in *NRF2*-silenced cancer cells restored BNIP3-mediated autophagy activation. A protective role of HIF-1α/BNIP3/autophagy from hypoxic cell death was verified by using an autophagy inhibitor; chloroquine treatment substantially reduced cell viability in hypoxic control cells, whereas viability of *NRF2*-silenced cells, which was significantly lower than control cells, was not affected by chloroquine treatment. These results suggest that the hypoxia-induced autophagy is impaired by *NRF2*-silencing and provides further evidence for the role of HIF-1α/BNIP3-mediated autophagy in adaptive survival of cancer cells.

**Fig. 7:**
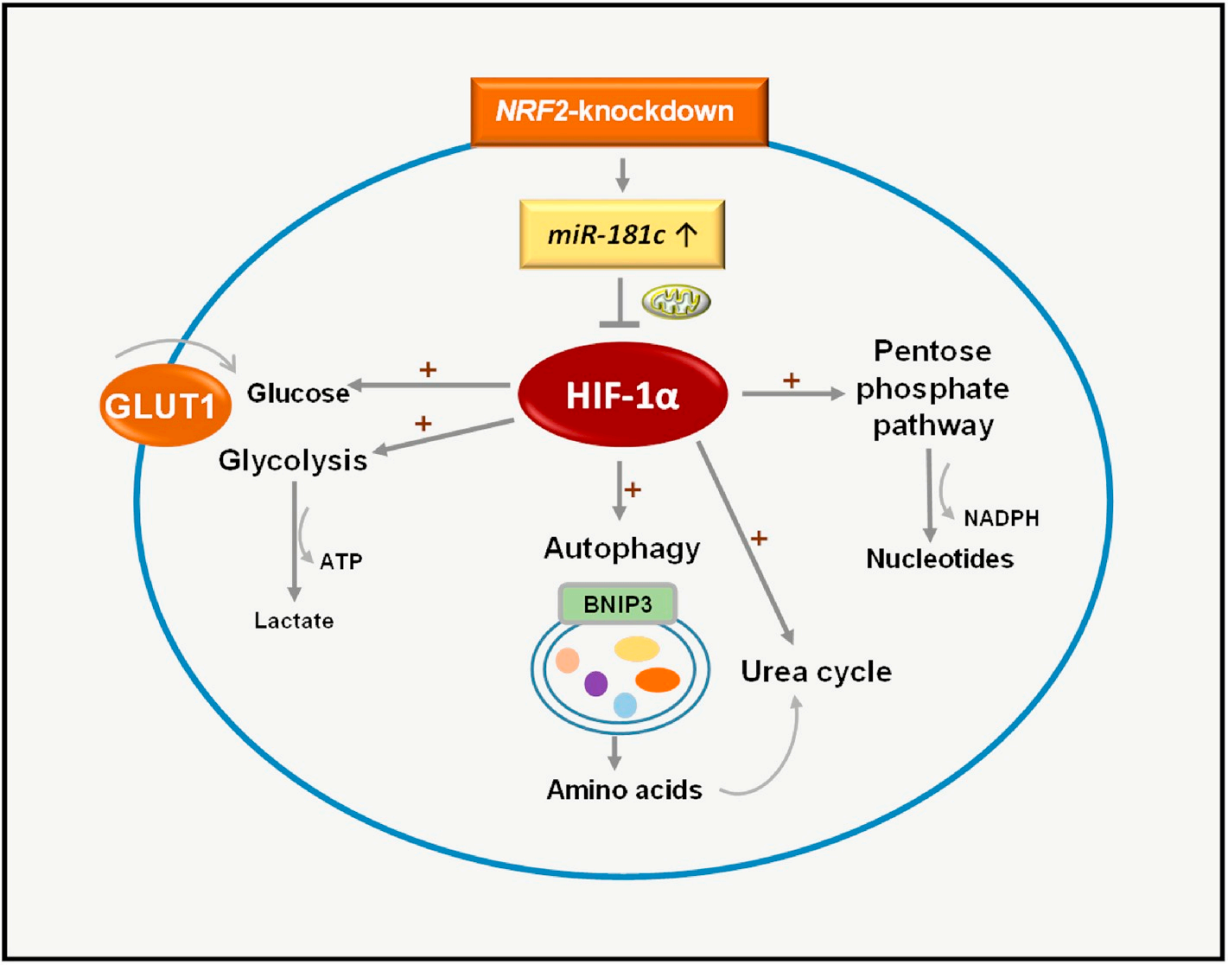
**Effects of *NRF2*-silencing on adaptive response to hypoxia in cancer cells**. *NRF2*-knockdown elevates *miR-181c* level, which leads to the inhibition of HIF-1α-mediated adaptive metabolic changes for glycolysis, PPP, and BNIP3-mediated autophagy activation under hypoxic condition.

Taken together, our results demonstrate that hypoxia-induced metabolic changes to glycolysis, PPP, and autophagy are suppressed by NRF2 inhibition through the blockage of HIF-1α accumulation and, further, suggest that elevation of *miR-181c* levels is a molecular link to connect NRF2 to HIF-1α dysregulation (Fig. 7). Thus, NRF2 and *miR-181c* could be effective targets to counteract HIF-1α-orchestrated metabolic adaptation of hypoxic cancer cells.

## Source of funding

This study was financially supported by a grant from the National Research Foundation of Korea (NRF) funded by the Korea government (MSIP) (2018R1A2A1A05078894, 2015R1A2A1A10054384, and 2018R1A6A1A03025108). This study was also supported by the BK21Plus grant of NRF funded by Korean government (22A20130012250).

## Disclosure

None.

## Conflict of interest

The authors declare that they have no conflict of interest.
