# Supplementary material for: Impairment of HIF-1α-mediated metabolic adaption by NRF2-silencing in breast cancer cells
Source: Redox Biol. 2019 May 2;24:101210. doi: 10.1016/j.redox.2019.101210 (PMC6514540; doi:10.1016/j.redox.2019.101210)
Supplement: Multimedia component 1 [file mmc1.docx]

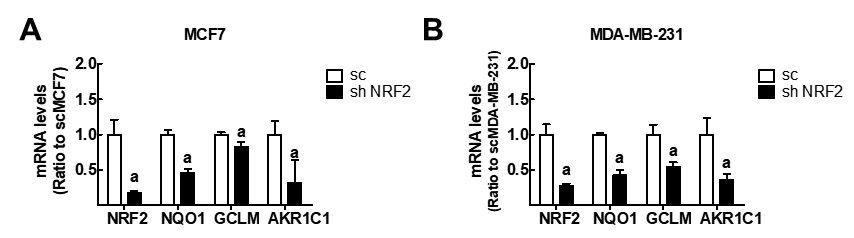


**Suppl. Fig. S1. Establishment of *NRF2*-silenced (shNRF2) breast cancer cell lines**

(A) Transcript levels for NRF2 and its target genes including NQO1, GCLM and AKR1C1 were measured in the control scMCF7 and shNRF2-MCF7 cells (B) Transcript levels for NRF2 and target genes in the scMDA-MB-231 and shNRF2-MDA-MB-231 cells. Data are expressed as means ± SD of three experiments. ^a^P <0.05 as compared to corresponding control cell line.


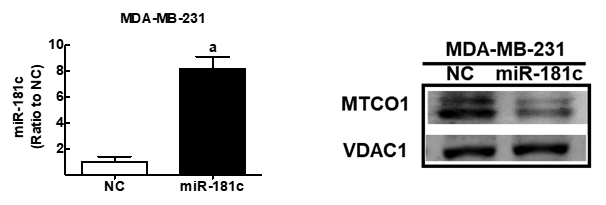


**Suppl. Fig. S2. Establishment of *miR-181c*-expressing breast cancer cells.**

Levels of *miR-181c* and MTCO1were measured in *miR-181c*-expressing MDA-MB-231 cells. Data are expressed as means ± SD of three experiments ^a^P <0.05 as compared to the nonspecific RNA-transfected control cells (NC).

**Suppl. Fig. S3. Reduced BNIP3 expression in *NRF2*-silenced MDA-MB-231 (shNRF2).** BNIP3 mRNA levels were assessed in *NRF2*-silenced MDA-MB-231 cells following hypoxia incubation for 24 h .


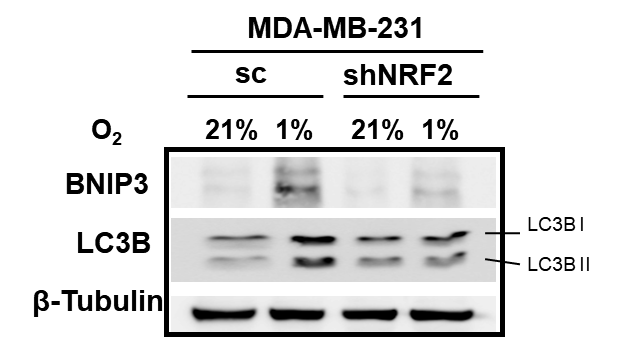


**Suppl. Fig. S4. Reduced autophagy markers expression in *NRF2*-silenced MDA-MB-231 (shNRF2).** The protein levels of BNIP3 and LC3B were measured following hypoxia-incubation for 24 h.

**Suppl. Fig. S5. HIF-1α-dependent elevation of BNIP3 mRNA in hypoxic MDA-MB-231 cells.**

BNIP3 mRNA levels were determined in hypoxic MDA-MB-231 cells following HIF-1α specific siRNA transfection.


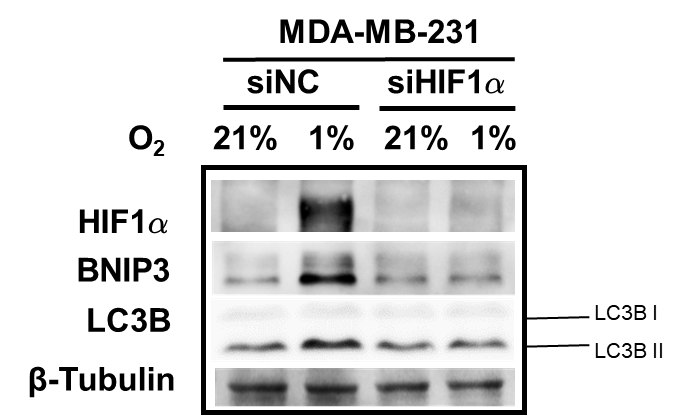


**Suppl. Fig. S6. HIF-1α-dependent autophagy markers expression in hypoxic MDA-MB-231 cells.**

The protein levels of BNIP3 and LC3B were measured in hypoxic MDA-MB-231 cells following HIF-1α specific siRNA transfection.


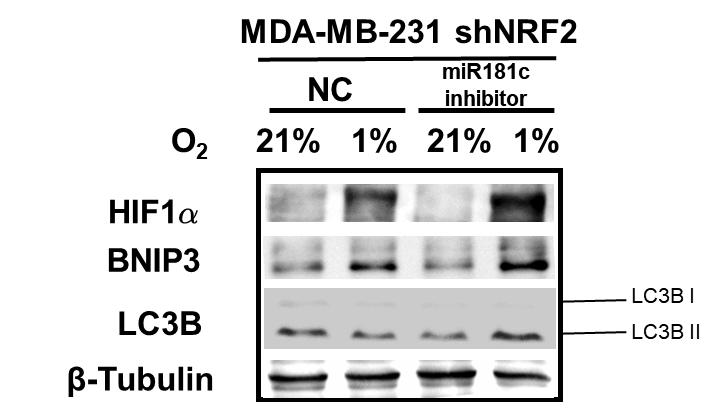


**Suppl. Fig. S7. Restoration of autophagy markers in hypoxic MDA-MB-231 cells following miR-181c inhibitor treatment.**

The protein levels of HIF-1α, BNIP3 and LC3B were measured in hypoxic MDA-MB-231 cells following miR-181c inhibitor transfection.
